# Lithium and Valproate Levels Do Not Correlate with Ketamine's Antidepressant Efficacy in Treatment-Resistant Bipolar Depression

**DOI:** 10.1155/2015/858251

**Published:** 2015-06-07

**Authors:** Annie J. Xu, Mark J. Niciu, Nancy B. Lundin, David A. Luckenbaugh, Dawn F. Ionescu, Erica M. Richards, Jennifer L. Vande Voort, Elizabeth D. Ballard, Nancy E. Brutsche, Rodrigo Machado-Vieira, Carlos A. Zarate

**Affiliations:** ^1^New York Medical College, 40 Sunshine Cottage Road, Valhalla, NY 10595, USA; ^2^National Institute of Mental Health, National Institutes of Health, Experimental Therapeutics and Pathophysiology Branch, 10 Center Drive, Building 10/CRC, Bethesda, MD 20892, USA; ^3^Massachusetts General Hospital, Depression Clinical & Research Program, 1 Bowdoin Square, 6th Floor, Boston, MA 02114, USA; ^4^Department of Psychiatry & Psychological Services, Mayo Clinic, 200 First Street SW, Rochester, MN 55905, USA

## Abstract

Ketamine and lithium both inhibit glycogen synthase kinase 3. In addition, lithium and ketamine have synergistic antidepressant-like effects at individually subeffective doses in rodents. We hypothesized that ketamine's antidepressant effects would be improved by therapeutic doses of lithium versus valproate and that serum lithium levels would positively correlate with ketamine's antidepressant efficacy. Thirty-six patients with treatment-resistant bipolar depression maintained on therapeutic-dose lithium (*n* = 23, 0.79 ± 0.15 mEq/L) or valproate (*n* = 13, 79.6 ± 12.4 mg/mL) received 0.5 mg/kg ketamine infusion in a randomized, double-blind, placebo-controlled, crossover trial. The primary depression outcome measure—the Montgomery-Åsberg Depression Rating Scale (MADRS)—was assessed before infusion and at numerous postinfusion time points. Both lithium (*F*_1,118_ = 152.08, *p* < 0.001, and *d* = 2.27) and valproate (*F*_1,128_ = 20.12, *p* < 0.001, and *d* = 0.79) significantly improved depressive symptoms, but no statistically significant difference was observed between mood stabilizer groups (*F*_1,28_ = 2.51, *p* = 0.12, and *d* = 0.60). Serum lithium and valproate levels did not correlate with ketamine's antidepressant efficacy. Although the study was potentially underpowered, our results suggest that lithium may not potentiate ketamine's antidepressant efficacy in treatment-resistant bipolar depression.

## 1. Introduction

In bipolar disorder (BD), major depressive episodes are more prevalent and disabling than the* sine qua non* of the disorder, (hypo)mania [1]. The depressive phase of the illness is also often more difficult to treat, and few United States Food and Drug Administration- (FDA-) approved options exist. As a result, in treatment-refractory bipolar depression, polypharmacy is common and often comes with a high side effect burden, for example, sedation, obesity, and metabolic abnormalities [2, 3]. A critical need exists for additional medication options with alternative mechanisms of action to treat refractory bipolar depression. We and others have demonstrated that subanesthetic dose ketamine—an N-methyl-*D*-aspartate (NMDA) receptor antagonist—has rapid-acting antidepressant and anxiolytic effects when used adjunctively to standard mood stabilizers, including therapeutic-dose lithium and valproate [4–6]. A single dose of ketamine also did not cause antidepressant-induced (hypo)mania in a combined sample from our two randomized, placebo-controlled, adjunctive ketamine trials [7]. This supports ketamine's safety and tolerability in potentially vulnerable patients with BD.

Glycogen synthase kinase 3 (GSK3) has been linked to the neuropathophysiology and treatment of mood disorders and schizophrenia [8]. Lithium both directly and indirectly inhibits GSK3 [9]. Lithium-induced GSK3 inhibition is dose-dependent, with an enzyme inhibition constant (Ki) of 1-2 mM (serum therapeutic range 0.6–1.2 mM) [10, 11] due to competition for magnesium [12] (magnesium is also the NMDA receptor pore block that must be removed for channel flux). Like lithium, ketamine has also been shown to inhibit GSK3 by increasing serine phosphorylation [13]. In rodents, the combination of low (subeffective) doses of NMDA receptor antagonists (including ketamine) and lithium has been reported to have synergistic antidepressant effects, as assessed via the forced swim test [14]. Finally, GSK3 inhibition by lithium and a selective GSK3 inhibitor (SB 216763) potentiated the antidepressant, synaptogenic, and electrophysiological effects of subthreshold dose (1 mg/kg) ketamine; these effects included mammalian target of rapamycin (mTOR) activation, increased excitatory postsynaptic currents (EPSCs), and dendritic spine morphogenesis [15].

Based on these preclinical observations, we hypothesized that the antidepressant effect of ketamine in bipolar depression would be synergized by lithium but not valproate, which likely exerts its mood-stabilizing properties predominately through GSK-independent mechanisms [16]. In addition, we hypothesized that greater antidepressant improvement would be associated with higher serum levels of lithium due to these putative synergistic inhibitory effects on GSK3. Finally, we hypothesized that lithium-treated subjects would have fewer dissociative and/or psychotomimetic side effects from ketamine than valproate-treated subjects.

## 2. Materials and Methods

All patients (*n* = 36) were admitted to the Inpatient Mood and Anxiety Disorder Research Unit at the National Institute of Mental Health (Bethesda, MD, USA). All participants provided written informed consent as approved by the NIH Combined Neuroscience Institutional Review Board (ClinicalTrials.gov Identifier: NCT00088699, NIH Protocol ID number: 04-M-0222, substudy 2). All subjects met DSM-IV criteria for BD (combined BD I and II) without psychotic symptoms based on clinical assessment and confirmed by structured diagnostic interview. Treatment-refractoriness was confirmed by nonresponse to at least one antidepressant (as confirmed by the modified Antidepressant Treatment History Form [17]). Lithium-maintained (*n* = 23) and valproate-maintained (*n* = 13) subjects had 9.36 ± 4.90 and 10.67 ± 2.08 lifetime adequate antidepressant trials, respectively (Table 1). Subjects had a Montgomery-Åsberg Depression Rating Scale (MADRS) score ≥20 at screening and at the start of each infusion. Exclusion criteria included current psychotic features, a diagnosis of schizophrenia or any other psychotic disorder, or active drug or alcohol abuse or dependence as defined by the fourth iteration of the Diagnostic and Statistical Manual of Mental Disorders (DSM-IV).

All data were secondarily analyzed from our double-blind, placebo-controlled, crossover (two weeks between infusions) add-on studies with a single subanesthetic dose (0.5 mg/kg) ketamine infusion or placebo over 40 minutes. The safety, tolerability, and efficacy data have been reported previously [4, 5]. All patients were maintained on therapeutic blood levels of either lithium (0.6–1.2 mEq/L) or valproate (50–125 mg/mL) for at least four weeks prior to the first ketamine infusion without additional standing or as-needed psychotropic medication administration. If applicable, adjunctive medications, for example, second-generation antipsychotics and antidepressants, were tapered off and discontinued for at least two weeks (five weeks for fluoxetine) before the first ketamine infusion. Therapeutic levels of lithium and valproate were confirmed during screening and also measured on the morning of each ketamine infusion; the latter levels (blood levels assessed the same day as the ketamine infusion) were used in this analysis. Change in MADRS score was assessed at 40, 80, 120, and 230 minutes after ketamine infusion, as well as one, two, three, seven, 10, and 14 days after infusion. Side effects were also measured at these time points via the Clinician-Administered Dissociative States Scale (CADSS) and the Brief Psychiatric Rating Scale (BPRS; the present analysis used data from the positive symptom portion of the BPRS only).

To examine differences in antidepressant response to ketamine based on mood stabilizer, a linear mixed model with restricted maximum likelihood estimation was used with time as a repeated measures factor and mood stabilizer (lithium versus valproate) and drug (ketamine versus placebo) as fixed factors. Our outcome measure for antidepressant response was total MADRS score. This was a full factorial model with a covariate of baseline MADRS and a first-order autoregressive covariance structure. In order to examine ketamine's side effect profile based on mood stabilizer, a linear mixed model with restricted maximum likelihood estimation (and the same repeated and fixed factors) was used with total CADSS and BPRS positive symptom scores as outcome measures and baseline CADSS and BPRS positive symptom scores as covariates, respectively.

The relationship between same-day mood stabilizer blood level and percent change in MADRS score from baseline was examined via bivariate correlational analyses. We used 230 minutes, one day, and seven days after infusion to contrast ketamine's same-day/hyperacute (230 min), next-day/acute (day 1), and sustained (day 7) antidepressant effects. These time points have been used in other published correlational analyses from our group [18–20]. Additional bivariate correlations were run with same-day mood stabilizer blood levels and change in CADSS and BPRS positive symptom scores from baseline to 40 minutes; this time point reflected when the ketamine infusion ended as well as when the greatest dissociative and psychotic-like adverse effects were likely to occur. Significance was evaluated at *p* < 0.05, two-tailed.

## 3. Results

This combined sample comprised 36 subjects with BD. Twenty-three and 13 subjects were maintained on therapeutic doses of lithium or valproate, respectively. No significant differences were observed for any of the demographic and clinical factors examined (*p* > 0.05, Table 1). On the day of the ketamine infusion, group lithium levels were 0.79 ± 0.15 mEq/L, and group valproate levels were 79.6 ± 12.4 mg/mL.

**Figure 1:**
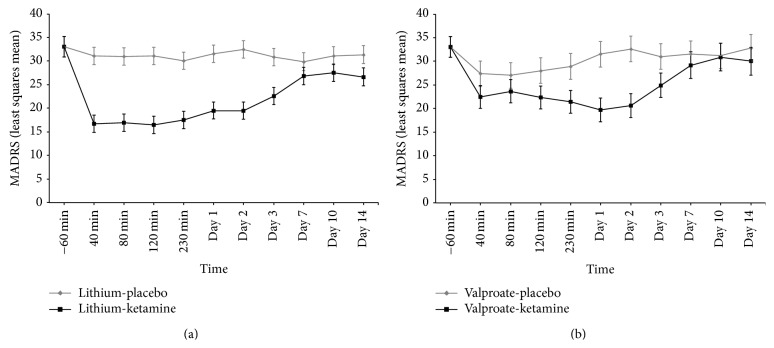
Rapid and sustained antidepressant effects of ketamine in treatment-resistant bipolar depressed patients maintained on therapeutic-dose lithium and valproate. Twenty-three subjects with treatment-resistant bipolar disorder (BD) receiving lithium (a) and 13 receiving valproate (b) currently experiencing a major depressive episode were randomized to either subanesthetic dose ketamine (0.5 mg/kg × 40 min) or placebo infusion in a randomized, placebo-controlled, crossover trial. Ketamine had antidepressant efficacy in both lithium-maintained and valproate-maintained patients (drug-by-mood stabilizer interaction (*F*_1,125_ = 8.26, *p* = 0.005)) but there was no statistically significant antidepressant difference between lithium and valproate (*F*_1,28_ = 2.51, *p* = 0.12, and *d* = 0.60).

We first used a linear mixed model to examine the antidepressant effect of ketamine based on total MADRS score in subjects taking lithium versus valproate (Figure 1). The model showed a significant drug-by-mood stabilizer interaction (*F*_1,125_ = 8.26, *p* = 0.005), but no three-way interaction with time (*F*_9,306_ = 0.90, *p* = 0.52). Depressive symptoms improved significantly in subjects receiving lithium (*F*_1,118_ = 152.08, *p* < 0.001, and *d* = 2.27) or valproate (*F*_1,128_ = 20.12, *p* < 0.001, and *d* = 0.79), but no statistically significant difference was noted between mood stabilizer groups (*F*_1,28_ = 2.51, *p* = 0.12, and *d* = 0.60). In our linear mixed model that examined the side effects of ketamine using total CADSS score, we found no significant drug-by-mood stabilizer effect (*F*_1,171_ = 0.39, *p* = 0.54) and no three-way interaction with time (*F*_9,360_ = 0.18, *p* = 1.00). In the linear mixed model using total BPRS positive symptom score as an outcome measure, we likewise found no drug-by-mood stabilizer effect (*F*_1,147_ = 0.034, *p* = 0.85) or three-way interaction with time (*F*_9,342_ = 0.088, *p* = 1.00).

**Figure 2:**
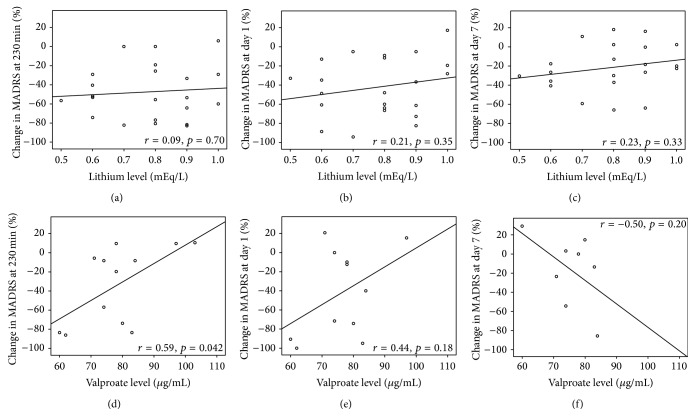
Therapeutic serum levels of lithium and valproate did not correlate with ketamine's antidepressant efficacy. Treatment-resistant patients with bipolar disorder (BD) currently experiencing a major depressive episode were maintained on therapeutic but subeffective serum levels of lithium or valproate for at least four weeks. Mean same-day pre-ketamine lithium (*n* = 22) and valproate (*n* = 12) levels were 0.79 ± 0.15 mEq/L and 79.6 ± 12.4 mg/mL, respectively. These levels correlated with ketamine's antidepressant efficacy at three time points: 230 minutes (same-day/hyperacute), day one (next-day/acute), and day seven (sustained) after ketamine infusion. Serum lithium levels did not correlate with ketamine's antidepressant efficacy at these three time points (a). Serum valproate levels positively correlated with ketamine's antidepressant efficacy at 230 minutes after infusion, *r* = 0.59, *p* = 0.04, but this did not survive adjustment for multiple comparisons (*p*_adjusted_ = 0.12) (b).

We next performed Pearson correlations to examine the relationship between drug blood levels and percent change in MADRS score from baseline to 230 minutes, one day, and seven days after ketamine infusion. Two patients dropped out before receiving ketamine but after receiving their first placebo infusion, one receiving lithium and one receiving valproate; thus, 21 lithium- and 12 valproate-treated patients were available for the bivariate correlational analyses (Figure 2). For valproate, we found a significant positive correlation at 230 minutes (*r* = 0.59, *p* = 0.04), but this correlation did not survive adjustment for multiple comparisons (*p*_adjusted_ = 0.12). Correlations for day 1 (*r* = 0.44, *p* = 0.18) and day 7 (*r* = −0.50, *p* = 0.20) were not significant. Same-day pre-ketamine infusion lithium levels also did not correlate with ketamine's antidepressant efficacy at the indicated time points (230 minutes: *r* = 0.09, *p* = 0.70; day 1: *r* = 0.21, *p* = 0.35; day 7: *r* = 0.26, *p* = 0.33). We again performed Pearson correlations with same-day mood stabilizer level and CADSS and BPRS positive symptom score change immediately at the end of infusion. Again, no significant correlations emerged in this analysis.

## 4. Discussion

We observed a significant interaction between drug and mood stabilizer in this sample of 36 subjects with treatment-resistant bipolar depression maintained on therapeutic-dose lithium (*n* = 23) or valproate (*n* = 13). Ketamine's antidepressant effect size relative to placebo was larger for lithium (*d* = 2.27) than valproate (*d* = 0.79), but no statistically significant difference was observed between these two agents. As observed in our prior studies of ketamine in bipolar depression, the antidepressant effects of a single subanesthetic dose ketamine infusion are transient and failed to separate from placebo by three days after infusion. After correcting for multiple comparisons, we found that pre-ketamine infusion same-day levels of lithium or valproate did not correlate with ketamine's antidepressant effects. No significant associations were observed between dissociative or psychotomimetic side effects and mood stabilizer in either the linear mixed model (type) or correlational analyses (same-day serum levels).

As noted above, GSK3 inhibition appears to be a central mood-stabilizing mechanism; other potential mechanisms include inositol monophosphatase inhibition [21], decreased membrane associated protein kinase C [22], increased protein kinase C-alpha activity [23], and proteasomal inhibition [24]. Valproate also has several putative mechanisms of action, including voltage-gated sodium channel inhibition [25], increased gamma-aminobutyric acid (GABA) [26], and GSK3 beta inhibition* in vitro* [27]; however, histone deacetylase (HDAC) inhibition seems to be its major mechanism of action as a mood stabilizer [28]. As demonstrated in preclinical models of neuropsychiatric disorders, GSK3 inhibition by lithium and HDAC inhibition by valproate initiate numerous downstream neuroprotective cascades, including increased neurotrophin expression/secretion (e.g., of brain-derived neurotrophic factor (BDNF)). In addition, preclinical studies found that the combination of lithium and valproate enhanced neuroprotection by synergistically inhibiting GSK3 [29].

Although we initially hypothesized that the lithium group would have greater antidepressant improvement due to lithium and ketamine's convergence on GSK3 inhibition, the lack of between-group differences between the lithium- and valproate-treated groups may be explained by a greater-than-hypothesized overlap in their mechanism(s) of action. In addition, studies have shown that both lithium and valproate reverse or minimize impaired neuroplasticity in BD; for instance, lithium restores mitochondrial dysfunction, increases central N-acetylaspartate levels, and increases cortical gray matter volume [30–32]. Other studies found that BD subjects who respond to ketamine have differential mitochondrial *β*-oxidation of fatty acids and, in preclinical studies, ketamine was found to stimulate the release of BDNF and concomitant synaptic plasticity [33–35]. Nevertheless, most of the above discoveries were identified in preclinical models of depression and/or small clinical samples, so their relevance to a heterogeneous, highly prevalent clinical disorder like bipolar depression is tentative.

Several possibilities might explain the lack of correlation between serum lithium levels and ketamine's antidepressant effects. First, with only 23 subjects, our study may be underpowered to detect a small-to-medium potentiating effect of lithium on ketamine's antidepressant effects. In addition, BD is a heterogeneous disorder with different subtypes, and the breadth of the disorder might not have been reflected in our small sample, leading to potential type II (false negative) errors. Second, in their rodent study, Liu and colleagues [15] showed that the combination of subeffective doses of ketamine and lithium had synergistic antidepressant effects equivalent to those of higher-dose ketamine. However, this may occur only at subtherapeutic levels of lithium and/or ketamine; at the higher doses used in this study, GSK3 may already be maximally inhibited. Third, the patients in our study were on steady-state therapeutic levels of either lithium or valproate for at least four weeks prior to their first infusion. In contrast, lithium was given acutely in the preclinical studies, suggesting that it may not synergize with ketamine when taken chronically, due to transcriptional/translational effects on GSK3 and/or downstream neurotrophins such as BDNF [36, 37]. Fourth, the patients in our study were a highly treatment-refractory population including some subjects who had not previously responded to lithium; in the preclinical studies, the rodents were treatment-naive. Thus, lithium and ketamine may only work synergistically in lithium-*responsive* patients. Fifth, as alluded to above, there may be metabolomic differences between humans and rodents that explain the lack of synergistic effects between ketamine and lithium and that have been associated with clinical nonresponse. These include increased serum levels of select ketamine metabolites like (2S,5S;2R,5R)-hydroxynorketamine [38] and altered mitochondrial *β*-oxidation of select fatty acids [33]. Finally, the wash-out period of two weeks (five for fluoxetine) and prior treatment with antipsychotics or antidepressants could still have affected outcome in this study given that chronic administration of antidepressants/antipsychotics may affect cycling and, in some patients, these effects persist beyond the medication's half-life [39, 40].

## 5. Conclusions

In conclusion, ketamine had rapid antidepressant effects in treatment-resistant patients with bipolar depression maintained on therapeutic levels of either lithium or valproate. Although the effect size was medium (*d* = 0.60) in a small sample (*n* = 36), no statistically significant antidepressant difference emerged between medications, and serum levels for both medications did not correlate with antidepressant efficacy at three time points. In the context of a disorder as heterogeneous as bipolar depression, future studies should define and characterize (both clinically and biologically) more homogeneous subgroups based on antidepressant response to adjunctive ketamine.

## Supplementary Material

National Institute of Mental Health Ketamine Bipolar Depression Clinical Trial Schematic.

## Figures and Tables

**Table 1:** Demographic and clinical characteristics of subjects with treatment-resistant bipolar depression maintained on therapeutic dose lithium (*n* = 23) and valproate (*n* = 13).

	Lithium (*n* = 23)	Valproate (*n* = 13)	*p* value
	Mean (SD)	Mean (SD)	

Age, years	45.04 (12.22)	49.62 (8.42)	0.24
Length of illness, years	26.96 (11.45)	32.00 (8.41)	0.17
Length of current depressive episode, months	17.71 (23.05)	16.00 (14.94)	0.81
Total lifetime antidepressant trials	9.36 (4.90)	10.67 (2.08)	0.67
Clinical ratings (at baseline)			
HAM-D	21.36 (4.18)	21.00 (3.16)	0.80
CADSS	2.50 (4.81)	2.33 (3.89)	0.92
HAM-A	21.00 (4.21)	21.91 (3.75)	0.55

	*n* (%)	*n* (%)	

Bipolar I disorder	14 (60.9%)	7 (53.8%)	0.74
Education			0.18
Prehigh school	0 (0.0%)	0 (0.0%)	
High school	4 (17.4%)	0 (0.0%)	
Some college	11 (47.8%)	4 (30.8%)	
College	4 (17.4%)	4 (30.8%)	
Graduate/professional	4 (17.4%)	5 (38.5%)	
Sex, female	15 (65.2%)	6 (46.2%)	0.31
Lifetime diagnosis			
Anxiety disorder	14 (60.9%)	5 (38.5%)	0.30
Alcohol use disorder	12 (52.2%)	7 (53.8%)	1.00
Substance use disorder (nonnicotine)	9 (39.1%)	8 (61.5%)	0.30
Family history			
Alcohol use disorder, 1st-degree relative	6 (26.1%)	7 (53.8%)	0.15
Alcohol use disorder, 2nd-degree relative	15 (65.2%)	5 (38.5%)	0.17
Mood disorder	20 (87.0%)	12 (92.3%)	1.00
Anxiety disorder	7 (30.4%)	2 (15.4%)	0.12
Suicide attempt	6 (26.1%)	4 (30.8%)	0.76
Lifetime history			
Suicide attempt	10 (43.5%)	8 (61.5%)	0.18
Abuse			
Physical	3 (13.0%)	3 (23.1%)	0.58
Sexual	6 (26.1%)	5 (38.5%)	0.46

HAM-D: Hamilton Depression Rating Scale; CADSS: Clinician-Administered Dissociative States Scale; HAM-A: Hamilton Rating Scale for Anxiety.

## References

[1] Baldessarini R. J., Vieta E., Calabrese J. R., Tohen M., Bowden C. L. (2010). Bipolar depression: overview and commentary. *Harvard Review of Psychiatry*.

[2] Tirupati S., Chua L.-E. (2007). Obesity and metabolic syndrome in a psychiatric rehabilitation service. *Australian and New Zealand Journal of Psychiatry*.

[3] Maayan L., Correll C. U. (2011). Weight gain and metabolic risks associated with antipsychotic medications in children and adolescents. *Journal of Child and Adolescent Psychopharmacology*.

[4] Diazgranados N., Ibrahim L., Brutsche N. E. (2010). A randomized add-on trial of an N-methyl-D-aspartate antagonist in treatment-resistant bipolar depression. *Archives of General Psychiatry*.

[5] Zarate C. A., Brutsche N. E., Ibrahim L. (2012). Replication of ketamine's antidepressant efficacy in bipolar depression: a randomized controlled add-on trial. *Biological Psychiatry*.

[6] Rybakowski J. K., Permoda-Osip A., Skibinska M., Adamski R., Bartkowska-Sniatkowska A. (2013). Single ketamine infusion in bipolar depression resistant to antidepressants: are neurotrophins involved?. *Human Psychopharmacology*.

[7] Niciu M. J., Luckenbaugh D. A., Ionescu D. F., Mathews D. C., Richards E. M., Zarate C. A. (2013). Subanesthetic dose ketamine does not induce an affective switch in three independent samples of treatment-resistant major depression. *Biological Psychiatry*.

[8] Hur E.-M., Zhou F.-Q. (2010). GSK3 signalling in neural development. *Nature Reviews Neuroscience*.

[9] Jope R. S. (2003). Lithium and GSK-3: one inhibitor, two inhibitory actions, multiple outcomes. *Trends in Pharmacological Sciences*.

[10] Klein P. S., Melton D. A. (1996). A molecular mechanism for the effect of lithium on development. *Proceedings of the National Academy of Sciences of the United States of America*.

[11] Stambolic V., Ruel L., Woodgett J. R. (1996). Lithium inhibits glycogen synthase kinase-3 activity and mimics wingless signalling in intact cells. *Current Biology*.

[12] Ryves W. J., Harwood A. J. (2001). Lithium inhibits glycogen synthase kinase-3 by competition for magnesium. *Biochemical and Biophysical Research Communications*.

[13] Beurel E., Song L., Jope R. S. (2011). Inhibition of glycogen synthase kinase-3 is necessary for the rapid antidepressant effect of ketamine in mice. *Molecular Psychiatry*.

[14] Ghasemi M., Raza M., Dehpour A. R. (2010). NMDA receptor antagonists augment antidepressant-like effects of lithium in the mouse forced swimming test. *Journal of Psychopharmacology*.

[15] Liu R.-J., Fuchikami M., Dwyer J. M., Lepack A. E., Duman R. S., Aghajanian G. K. (2013). GSK-3 inhibition potentiates the synaptogenic and antidepressant-like effects of subthreshold doses of ketamine. *Neuropsychopharmacology*.

[16] Atmaca M. (2009). Valproate and neuroprotective effects for bipolar disorder. *International Review of Psychiatry*.

[17] Sackeim H. A. (2001). The definition and meaning of treatment-resistant depression. *Journal of Clinical Psychiatry*.

[18] Niciu M. J., Luckenbaugh D. A., Ionescu D. F. (2014). Clinical predictors of ketamine response in treatment-resistant major depression. *Journal of Clinical Psychiatry*.

[19] Luckenbaugh D. A., Niciu M. J., Ionescu D. F. (2014). Do the dissociative side effects of ketamine mediate its antidepressant effects?. *Journal of Affective Disorders*.

[20] Lundin N., Niciu M., Luckenbaugh D. (2014). Baseline vitamin B12 and folate levels do not predict improvement in depression after a single infusion of ketamine. *Pharmacopsychiatry*.

[21] Vadnal R., Parthasarathy R. (1995). Myo-inositol monophosphatase: diverse effects of lithium, carbamazepine, and valproate. *Neuropsychopharmacology*.

[22] Manji H. K., Etcheberrigaray R., Chen G., Olds J. L. (1993). Lithium decreases membrane-associated protein kinase C in hippocampus: Selectivity for the *α* isozyme. *Journal of Neurochemistry*.

[23] Kirshenboim N., Plotkin B., Shlomo S. B., Kaidanovich-Beilin O., Eldar-Finkelman H. (2004). Lithium-mediated phosphorylation of glycogen synthase kinase-3*β* involves PI3 kinase-dependent activation of protein kinase C-*α*. *Journal of Molecular Neuroscience*.

[24] Shim M., Smart R. C. (2003). Lithium stabilizes the CCAAT/enhancer-binding protein a (C/EBP*α*) through a glycogen synthase kinase 3 (GSK3)-independent pathway involving direct inhibition of proteasomal activity. *The Journal of Biological Chemistry*.

[25] Stahl S. M. (2004). Anticonvulsants as mood stabilizers and adjuncts to antipsychotics: valproate, lamotrigine, carbamazepine, and oxcarbazepine and actions at voltage-gated sodium channels. *Journal of Clinical Psychiatry*.

[26] Emrich H. M., von Zerssen D., Kissling W., Möller H. J. (1981). On a possible role of GABA in mania. Therapeutic efficacy of sodium valproate. *Advances in Biochemical Psychopharmacology*.

[27] Chen G., Huang L.-D., Jiang Y.-M., Manji H. K. (1999). The mood-stabilizing agent valproate inhibits the activity of glycogen synthase kinase-3. *Journal of Neurochemistry*.

[28] Chiu C.-T., Wang Z., Hunsberger J. G., Chuang D.-M. (2013). Therapeutic potential of mood stabilizers lithium and valproic acid: beyond bipolar disorder. *Pharmacological Reviews*.

[29] Leng Y., Liang M. H., Ren M., Marinova Z., Leeds P., Chuang D.-M. (2008). Synergistic neuroprotective effects of lithium and valproic acid or other histone deacetylase inhibitors in neurons: Roles of glycogen synthase kinase-3 inhibition. *The Journal of Neuroscience*.

[30] Quiroz J. A., Gray N. A., Kato T., Manji H. K. (2008). Mitochondrially mediated plasticity in the pathophysiology and treatment of bipolar disorder. *Neuropsychopharmacology*.

[31] Kempton M. J., Geddes J. R., Ettinger U., Williams S. C. R., Grasby P. M. (2008). Meta-analysis, database, and meta-regression of 98 structural imaging studies in bipolar disorder. *Archives of General Psychiatry*.

[32] MacHado-Vieira R., Soeiro-De-Souza M. G., Richards E. M., Teixeira A. L., Zarate C. A. (2014). Multiple levels of impaired neural plasticity and cellular resilience in bipolar disorder: developing treatments using an integrated translational approach. *World Journal of Biological Psychiatry*.

[33] Villaseñor A., Ramamoorthy A., Silva dos Santos M. (2014). A pilot study of plasma metabolomic patterns from patients treated with ketamine for bipolar depression: evidence for a response-related difference in mitochondrial networks. *British Journal of Pharmacology*.

[34] Li N., Lee B., Liu R.-J. (2010). mTOR-dependent synapse formation underlies the rapid antidepressant effects of NMDA antagonists. *Science*.

[35] Autry A. E., Adachi M., Nosyreva E. (2011). NMDA receptor blockade at rest triggers rapid behavioural antidepressant responses. *Nature*.

[36] Cunha A. B. M., Frey B. N., Andreazza A. C. (2006). Serum brain-derived neurotrophic factor is decreased in bipolar disorder during depressive and manic episodes. *Neuroscience Letters*.

[37] Tramontina J. F., Andreazza A. C., Kauer-Sant'Anna M. (2009). Brain-derived neurotrophic factor serum levels before and after treatment for acute mania. *Neuroscience Letters*.

[38] Zarate C. A., Brutsche N., Laje G. (2012). Relationship of ketamine's plasma metabolites with response, diagnosis, and side effects in major depression. *Biological Psychiatry*.

[39] Calabrese J. R., Shelton M. D., Bowden C. L. (2001). Bipolar rapid cycling: focus on depression as its hallmark. *Journal of Clinical Psychiatry*.

[40] Thase M. E. (2012). Bipolar disorder maintenance treatment: monitoring effectiveness and safety. *The Journal of Clinical Psychiatry*.

