# Supplementary material for: Lithium and Valproate Levels Do Not Correlate with Ketamine's Antidepressant Efficacy in Treatment-Resistant Bipolar Depression
Source: Neural Plast. 2015 Jun 7;2015:858251. doi: 10.1155/2015/858251 (PMC4475570; doi:10.1155/2015/858251)

Supplementary Figure 1 – Study Design Schematic. During the initial screening phase, individuals with bipolar disorder (BD) I/II were initiated and titrated on lithium or valproate within the accepted therapeutic range (lithium: 0.6-1.2 mEq/L; valproate: 50-125 mg/ml) for at least four weeks. All other psychotropic medications were tapered and discontinued for at least two weeks (five weeks for fluoxetine). All subjects were then randomized and double-blinded to receive either 0.5mg/kg ketamine vs. saline placebo intravenously over 40 minutes in a counterbalanced cross-over design. Standardized rating scales (including the primary outcome measure, the Montgomery-Åsberg Depression Rating Scale (MADRS)) were measured at baseline (~60 minutes prior to ketamine infusion), at several post-infusion time points (+40, +80, +120, and +230 minutes) and on the following post-infusion days: 1(15), 2(16), 3(17), 7(21), 10(24) and 14(28) (days in parentheses correspond to the days following the second infusion). Notably, day 14 was also the day of the second infusion, and subjects needed to continue to meet all inclusion/exclusion criteria for continued participation, e.g. same-day depression severity and therapeutic dose mood stabilizer levels.

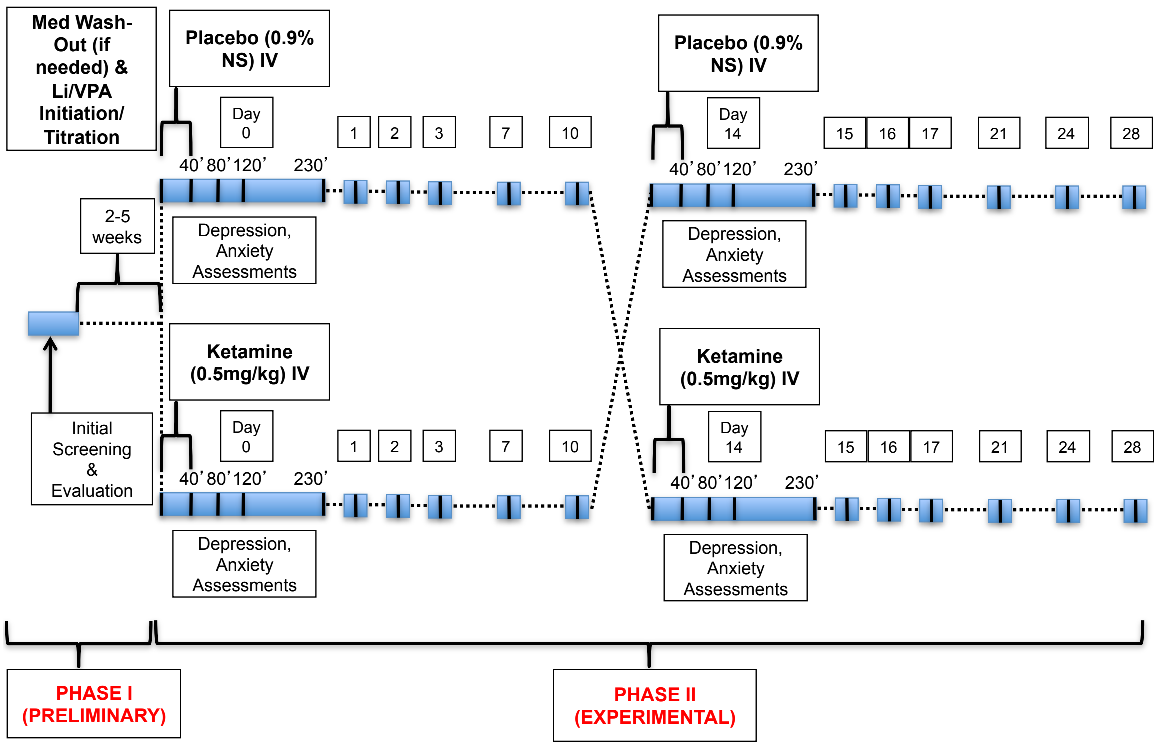

Supplement: Supplementary file 1 — National Institute of Mental Health Ketamine Bipolar Depression Clinical Trial Schematic. [file 858251.f1.pdf]
